# Supplementary material for: Transcriptomic Profiling of Peripheral Edge of Lesions to Elucidate the Pathogenesis of Psoriasis Vulgaris
Source: Int J Mol Sci. 2022 Apr 30;23(9):4983. doi: 10.3390/ijms23094983 (PMC9101153; doi:10.3390/ijms23094983)
Supplement: Supplementary file 1 [file ijms-23-04983-s001.zip › Supplementary_of_Transcriptomic_profiling_of_peripheral_edge_of_lesion_to_generate.pdf]

Supplementary Material

1. **Result**
   1.1. RNA sequencing

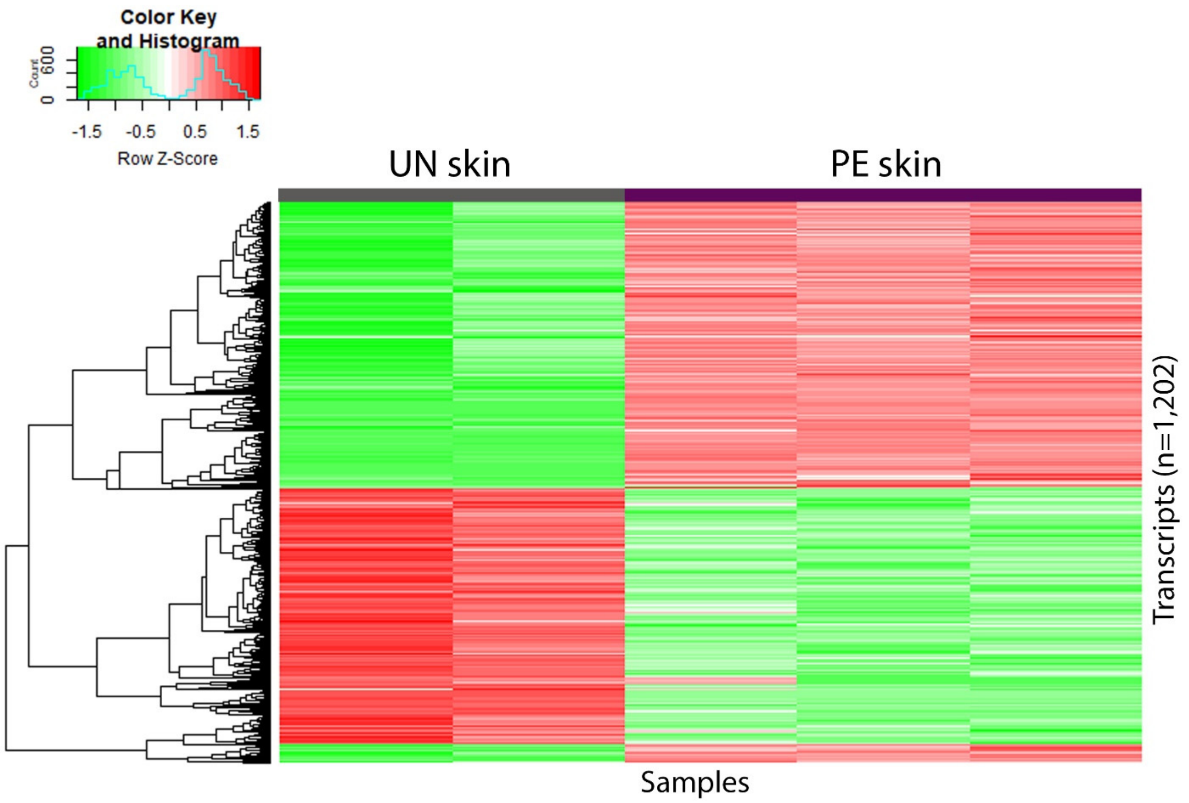

Supplementary Figure S1. The heat map of RNA-seq data.
Differentially altered transcripts between PE and UN skin (n=1,202) are shown in a heatmap image. The green color indicates low expression levels whereas red indicates high expression levels. The purple bar above the heatmap indicates PR skin and the grey bar above the heatmap indicates UN skin. PE skin, peripheral edge of lesional skin; UN skin, uninvolved skin.

1.2. DEGs analysis

Supplementary Table S1. The top-100 upregulated DEGs by Log 2FC.

	Gene symbol	Gene title	Molecule type*	Log 2FC	*p*-value	FDR
1	*A2M*	alpha-2-macroglobulin	transporter	15.982	8.60E-06	0.032
2	*H3C2*	H3 clustered histone 2	other	15.367	9.34E-05	0.037
3	*PLEC*	Plectin	other	15.295	1.36E-05	0.032
4	*ITSN2*	intersectin 2	other	14.968	3.20E-04	0.056
5	*UGGT1*	UDP-glucose glycoprotein glucosyltransferase 1	enzyme	14.906	1.08E-04	0.038
6	*STT3A*	STT3 oligosaccharyltransferase complex catalytic subunit A	enzyme	14.745	3.46E-03	0.127
7	*HERC6*	HECT and RLD domain containing E3 ubiquitin protein ligase family member 6	enzyme	14.724	1.12E-03	0.084
8	*DEFB4A/DEFB4B*	defensin beta 4A	other	14.690	1.40E-04	0.038
9	*CAND1*	cullin associated and neddylation dissociated 1	transcription regulator	14.654	8.54E-03	0.183
10	*HRNR*	Hornerin	other	14.597	1.34E-05	0.032
11	*IL36A*	interleukin 36 alpha	cytokine	14.572	1.34E-04	0.038
12	*SMARCA4*	SWI/SNF related, matrix associated, actin dependent regulator of chromatin, subfamily a, member 4	transcription regulator	14.517	2.63E-05	0.032
13	*CYFIP1*	cytoplasmic FMR1 interacting protein 1	translation regulator	14.500	2.82E-03	0.116
14	*HIPK3*	homeodomain interacting protein kinase 3	kinase	14.365	6.20E-03	0.161
15	*PLA2G4E-AS1*	PLA2G4E antisense RNA 1	other	14.274	8.59E-05	0.037
16	*TRIP12*	thyroid hormone receptor interactor 12	enzyme	14.146	4.66E-03	0.141
17	*CLIC1*	chloride intracellular channel 1	ion channel	14.091	4.11E-05	0.033
18	*CD164*	CD164 molecule	other	14.069	1.28E-03	0.087
19	*BZW1*	basic leucine zipper and W2 domains 1	translation regulator	14.025	6.55E-03	0.163
20	*NRBP1*	nuclear receptor binding protein 1	kinase	13.939	1.28E-04	0.038
21	*PSMD12*	proteasome 26S subunit, non-ATPase 12	other	13.697	8.81E-05	0.037
22	*EPB41L3*	erythrocyte membrane protein band 4.1 like 3	other	13.662	2.00E-03	0.103
23	*SCYL2*	SCY1 like pseudokinase 2	other	13.652	1.49E-04	0.039
24	*H2BC10*	H2B clustered histone 10	other	13.610	1.87E-04	0.044
25	*WDR75*	WD repeat domain 75	other	13.554	1.18E-03	0.085
26	*H4C13*	H4 clustered histone 13	other	13.550	1.08E-03	0.083
27	*EXT2*	exostosin glycosyltransferase 2	enzyme	13.432	4.74E-04	0.064
28	*H3C7*	H3 clustered histone 7	other	13.405	1.48E-03	0.091
29	*PLEKHG5*	pleckstrin homology and RhoGEF domain containing G5	other	13.319	2.03E-04	0.045
30	*DMXL1*	Dmx like 1	other	13.302	1.93E-03	0.103
31	*GBP6*	guanylate binding protein family member 6	enzyme	13.278	2.32E-03	0.109
32	*H3C11*	H3 clustered histone 11	other	13.099	4.59E-04	0.064
33	*H2AC14*	H2A clustered histone 14	other	13.092	4.20E-06	0.026
34	*SMC6*	structural maintenance of chromosomes 6	other	12.917	2.03E-04	0.045
35	*H2AC21*	H2A clustered histone 21	other	12.847	7.45E-04	0.072
36	*H2AC4*	H2A clustered histone 4	other	12.793	5.31E-04	0.065
37	*CD59*	CD59 molecule (CD59 blood group)	other	12.792	9.37E-04	0.080
38	*TMEM14EP*	transmembrane protein 14E, pseudogene	other	12.753	6.24E-04	0.069
39	*GLB1*	galactosidase beta 1	enzyme	12.717	1.27E-03	0.086
40	*CTNNBL1*	catenin beta like 1	other	12.703	2.09E-03	0.105
41	*GFPT1*	glutamine--fructose-6-phosphate transaminase 1	enzyme	12.637	3.91E-03	0.132
42	*H3C8*	H3 clustered histone 8	other	12.599	1.30E-04	0.038
43	*ZDHHC6*	zinc finger DHHC-type palmitoyltransferase 6	enzyme	12.565	4.94E-03	0.144
44	*IKBKB*	inhibitor of nuclear factor kappa B kinase subunit beta	kinase	12.437	2.28E-03	0.107
45	*GANAB*	glucosidase II alpha subunit	enzyme	12.412	2.17E-04	0.046
46	*TMEM184B*	transmembrane protein 184B	other	12.410	1.41E-03	0.089
47	*SEPTIN11*	septin 11	other	12.377	9.90E-04	0.081
48	*RMDN3*	regulator of microtubule dynamics 3	other	12.322	3.10E-03	0.122
49	*LPP*	LIM domain containing preferred translocation partner in lipoma	other	12.292	2.60E-03	0.113
50	*H2BC13*	H2B clustered histone 13	other	12.292	5.60E-03	0.152
51	*MCTP1*	multiple C2 and transmembrane domain containing 1	other	12.291	1.00E-03	0.081

52	*CAP1*	cyclase associated actin cytoskeleton regulatory protein 1	other	12.224	2.19E-03	0.105
53	*SLC6A9*	solute carrier family 6 member 9	transporter	12.169	3.08E-03	0.122
54	*SSR3*	signal sequence receptor subunit 3	other	12.124	1.38E-03	0.089
55	*SPRR2C*	small proline rich protein 2C (pseudogene)	other	12.058	9.68E-03	0.191
56	*TBC1D22A*	TBC1 domain family member 22A	other	12.048	3.29E-03	0.124
57	*VMP1*	vacuole membrane protein 1	other	12.040	8.84E-03	0.185
58	*ERCC3*	ERCC excision repair 3, TFIIH core complex helicase subunit	enzyme	12.024	1.44E-03	0.090
59	*DENND5A*	DENN domain containing 5A	other	11.952	3.25E-03	0.124
60	*H4C1*	H4 clustered histone 1	other	11.942	4.08E-03	0.133
61	*MPP1*	membrane palmitoylated protein 1	kinase	11.925	1.63E-03	0.094
62	*TRAPPC9*	trafficking protein particle complex 9	other	11.903	2.33E-03	0.109
63	*EDNRB*	endothelin receptor type B	G-protein coupled receptor	11.815	5.82E-03	0.156
64	*MYH11*	myosin heavy chain 11	other	11.751	6.25E-05	0.035
65	*CDR1*	cerebellar degeneration related protein 1	other	11.657	2.49E-03	0.112
66	*UTY*	ubiquitously transcribed tetratricopeptide repeat containing, Y-linked	enzyme	11.645	2.94E-03	0.120
67	*PISD*	phosphatidylserine decarboxylase	enzyme	11.566	1.93E-03	0.103
68	*RELCH*	RAB11 binding and LisH domain, coiled-coil and HEAT repeat containing	other	11.548	2.20E-03	0.105
69	*AHNAK*	AHNAK nucleoprotein	other	11.483	2.50E-03	0.112
70	*H2AC15*	H2A clustered histone 15	other	11.462	6.90E-03	0.166
71	*SNORA94*	small nucleolar RNA, H/ACA box 94	other	11.406	7.09E-03	0.167
72	*RBM5-AS1*	RBM5 antisense RNA 1	other	11.393	6.28E-03	0.161
73	*SLC24A1*	solute carrier family 24 member 1	transporter	11.355	7.95E-03	0.177
74	*TOP1MT*	DNA topoisomerase I mitochondrial	enzyme	11.254	8.62E-04	0.077
75	*EGFR-AS1*	EGFR antisense RNA 1	other	11.235	8.58E-03	0.184
76	*THRIL*	TNF and HNRNPL related immunoregulatory long non-coding RNA	other	11.196	6.76E-03	0.165
77	*UXS1*	UDP-glucuronate decarboxylase 1	enzyme	11.179	9.15E-03	0.189
78	*SMG5*	SMG5 nonsense mediated mRNA decay factor	other	11.140	1.00E-03	0.081
79	*FAM104A*	family with sequence similarity 104 member A	other	11.126	7.56E-03	0.173
80	*PCLO*	piccolo presynaptic cytomatrix protein	transporter	11.096	9.26E-03	0.189
81	*H4C5*	ankyrin 3	other	10.982	8.66E-04	0.077
82	*ANK3*	H4 clustered histone 5	other	10.982	6.31E-03	0.161
83	*SCARNA13*	small Cajal body-specific RNA 13	other	10.936	5.46E-06	0.026
84	*SNORD133*	small nucleolar RNA, C/D box 133	other	10.891	1.05E-03	0.082
85	*TWF1*	twinfilin actin binding protein 1	kinase	10.854	5.24E-03	0.148
86	*INSYN2A*	inhibitory synaptic factor 2A	other	10.841	8.15E-03	0.180
87	*TNIP3*	TNFAIP3 interacting protein 3	other	10.794	6.58E-03	0.163
88	*KIFAP3*	kinesin associated protein 3	other	10.753	1.78E-03	0.099
89	*H1-1*	H1.1 linker histone, cluster member	other	10.710	5.23E-03	0.148
90	*H3C14*	H3 clustered histone 14	other	10.708	1.47E-03	0.090
91	*SERPINB4*	serpin family B member 4	other	10.691	7.43E-03	0.172
92	*FBXO28*	F-box protein 28	other	10.682	4.89E-03	0.144
93	*CXCL8*	C-X-C motif chemokine ligand 8	cytokine	10.681	6.58E-04	0.070
94	*MPZL3*	myelin protein zero like 3	other	10.651	7.44E-04	0.072
95	*H3C3*	H3 clustered histone 3	other	10.623	4.60E-04	0.064
96	*PRKAG1*	protein kinase AMP-activated non-catalytic subunit gamma 1	kinase	10.622	1.06E-03	0.082
97	*AKAP1*	A-kinase anchoring protein 1	other	10.614	3.80E-03	0.131
98	*PSTPIP1*	proline-serine-threonine phosphatase interacting protein 1	other	10.612	9.05E-03	0.188
99	*RAB3GAP1*	RAB3 GTPase activating protein catalytic subunit 1	other	10.600	1.21E-03	0.085
100	*H2AC13*	H2A clustered histone 13	other	10.576	2.72E-05	0.032

Blue molecules are molecules that have previously been mentioned in psoriasis, either their mRNA or their proteins based on QIAGEN Knowledge Base as of October 2021.

*Molecule types are classified into complex, cytokine, enzyme, fusion gene/product, G-protein coupled receptor, group, growth factor, ion channel, kinase, ligand-dependent nuclear receptor, mature microRNA, peptidase, phosphatase, transcription regulator, translation regulator, transmembrane receptor and transporter. None of these were classified as other. DEGs, differentially expressed genes; FC, fold change.

Supplementary Table S2 The top-100 downregulated DEGs by log2FC.

	Gene symbol	Gene title	Molecule type*	Log 2FC	*p*-value	FDR
1	*ATP5F1A*	ATP synthase F1 subunit alpha	transporter	-27.343	7.380E-04	0.072
2	*SEPTIN5*	septin 5	enzyme	-25.527	1.410E-03	0.089
3	*KHNYN*	KH and NYN domain containing	other	-24.254	3.700E-03	0.130
4	*PLD3*	phospholipase D family member 3	enzyme	-23.010	5.630E-03	0.153
5	*FHL1*	four and a half LIM domains 1	other	-21.983	9.840E-03	0.193
6	*HEXA*	hexosaminidase subunit alpha	enzyme	-20.826	8.320E-03	0.181
7	*DNM2*	dynamin 2	enzyme	-19.584	7.500E-03	0.172
8	*TLE5*	TLE family member 5, transcriptional modulator	transcription regulator	-18.613	3.990E-03	0.132
9	*MYH14*	myosin heavy chain 14	enzyme	-16.846	1.460E-03	0.090
10	*GSN*	Gelsolin	other	-16.812	1.490E-03	0.091
11	*SPSB3*	splA/ryanodine receptor domain and SOCS box containing 3	other	-16.593	1.100E-03	0.084
12	*LCE1C*	late cornified envelope 1C	other	-16.153	1.620E-03	0.094
13	*LAMTOR4*	late endosomal/lysosomal adaptor, MAPK and MTOR activator 4	other	-16.058	9.610E-05	0.037
14	*SYT8*	synaptotagmin 8	transporter	-16.014	9.260E-03	0.189
15	*LTBP4*	latent transforming growth factor beta binding protein 4	growth factor	-15.985	2.600E-03	0.113
16	*ITGB4*	integrin subunit beta 4	transmembrane receptor	-15.717	3.270E-03	0.124
17	*DCTN1*	dynactin subunit 1	other	-15.591	3.700E-03	0.130
18	*RTN4*	reticulon 4	other	-15.532	3.930E-03	0.132
19	*CAMSAP3*	calmodulin regulated spectrin associated protein family member 3	other	-15.517	3.990E-03	0.132
20	*NFIX*	nuclear factor I X	transcription regulator	-15.266	5.070E-03	0.146
21	*CRAT*	carnitine O-acetyltransferase	enzyme	-15.226	4.210E-03	0.135
22	*RAB40C*	RAB40C, member RAS oncogene family	enzyme	-15.156	5.860E-03	0.156
23	*CCL27*	C-C motif chemokine ligand 27	other	-15.021	3.820E-03	0.131
24	*SIK1/SIK1B*	salt inducible kinase 1	kinase	-14.994	6.560E-03	0.163
25	*CAPG*	capping actin protein, gelsolin like	other	-14.970	2.080E-04	0.045
26	*EPN2*	epsin 2	other	-14.879	7.780E-03	0.175
27	*ARAP1*	ArfGAP with RhoGAP domain, ankyrin repeat and PH domain 1	other	-14.871	7.950E-03	0.177
28	*PDCD4*	programmed cell death 4	other	-14.858	7.870E-03	0.176
29	*XPO6*	exportin 6	other	-14.735	9.330E-03	0.189
30	*PODN*	Podocan	other	-14.705	9.560E-03	0.190
31	*TUBGCP2*	tubulin gamma complex associated protein 2	peptidase	-14.694	9.730E-03	0.192
32	*CIRBP*	cold inducible RNA binding protein	translation regulator	-14.658	9.860E-03	0.193
33	*TSPAN14*	tetraspanin 14	other	-14.640	9.940E-03	0.193
34	*ATP2B4*	ATPase plasma membrane Ca2+ transporting 4	transporter	-14.506	1.540E-03	0.092
35	*CNOT1*	CCR4-NOT transcription complex subunit 1	other	-14.414	8.590E-04	0.077
36	*WBP2*	WW domain binding protein 2	transcription regulator	-14.403	3.680E-03	0.129
37	*MAN2C1*	mannosidase alpha class 2C member 1	enzyme	-14.324	2.770E-03	0.115
38	*POLD2*	DNA polymerase delta 2, accessory subunit	enzyme	-14.293	6.080E-04	0.068
39	*MPST*	mercaptopyruvate sulfurtransferase	enzyme	-14.237	5.410E-03	0.150
40	*ACSS2*	acyl-CoA synthetase short chain family member 2	enzyme	-14.029	4.050E-03	0.133
41	*AP2A1*	adaptor related protein complex 2 subunit alpha 1	transporter	-13.944	4.740E-03	0.142
42	*SCRIB*	scribble planar cell polarity protein	other	-13.900	6.080E-03	0.159
43	*TAP2*	transporter 2, ATP binding cassette subfamily B member	transporter	-13.808	8.590E-03	0.184
44	*PHYHIP*	phytanoyl-CoA 2-hydroxylase interacting protein	other	-13.783	6.750E-03	0.165
45	*PLCH2*	phospholipase C eta 2	enzyme	-13.780	6.560E-03	0.163
46	*TMEM63B*	transmembrane protein 63B	ion channel	-13.617	6.260E-03	0.161
47	*TAX1BP1*	Tax1 binding protein 1	other	-13.600	3.020E-03	0.121
48	*PKM*	pyruvate kinase M1/2	kinase	-13.594	5.310E-03	0.149
49	*EPHX3*	epoxide hydrolase 3	enzyme	-13.472	8.680E-03	0.184
50	*PTBP1*	polypyrimidine tract binding protein 1	enzyme	-13.167	8.660E-03	0.184
51	*FCGRT*	Fc fragment of IgG receptor and transporter	transmembrane receptor	-13.037	6.690E-03	0.164
52	*MARCHF2*	membrane associated ring-CH-type finger 2	enzyme	-12.985	3.850E-03	0.132
53	*RPS3*	ribosomal protein S3	enzyme	-12.891	4.110E-03	0.134
54	*ZFYVE16*	zinc finger FYVE-type containing 16	other	-12.664	1.810E-03	0.100

55	*TPPP3*	tubulin polymerization promoting protein family member 3	other	-12.098	7.770E-03	0.175
56	*STUB1*	STIP1 homology and U-box containing protein 1	enzyme	-11.875	6.210E-03	0.161
57	*FBXO22*	F-box protein 22	enzyme	-11.708	6.990E-03	0.167
58	*MMP28*	matrix metallopeptidase 28	peptidase	-11.574	7.540E-03	0.173
59	*TMBIM6*	transmembrane BAX inhibitor motif containing 6	other	-11.570	8.740E-04	0.077
60	*STK25*	serine/threonine kinase 25	kinase	-10.319	3.940E-03	0.132
61	*TAGLN2*	transgelin 2	other	-9.939	8.500E-03	0.183
62	*CFD*	complement factor D	peptidase	-9.500	8.790E-03	0.185
63	*ESRRA*	estrogen related receptor alpha	transcription regulator	-9.499	9.520E-03	0.190
64	*CYP1A1*	cytochrome P450 family 1 subfamily A member 1	enzyme	-9.340	4.600E-04	0.064
65	*LORICRIN*	loricrin cornified envelope precursor protein	other	-9.244	8.540E-04	0.077
66	*ABHD16A*	abhydrolase domain containing 16A, phospholipase	enzyme	-9.026	6.300E-03	0.161
67	*ATP6V0B*	ATPase H+ transporting V0 subunit b	transporter	-8.566	2.600E-03	0.113
68	*HS3ST6*	heparan sulfate-glucosamine 3-sulfotransferase 6	enzyme	-8.404	1.380E-03	0.089
69	*RBM10*	RNA binding motif protein 10	other	-8.066	3.500E-03	0.127
70	*EEF1D*	eukaryotic translation elongation factor 1 delta	translation regulator	-7.844	2.260E-03	0.107
71	*CAMTA2*	calmodulin binding transcription activator 2	transcription regulator	-7.756	4.210E-03	0.135
72	*ALDOA*	aldolase, fructose-bisphosphate A	enzyme	-7.337	5.810E-03	0.156
73	*SOD3*	superoxide dismutase 3	enzyme	-7.189	1.040E-03	0.082
74	*RPL37A*	ribosomal protein L37a	other	-6.625	1.230E-04	0.038
75	*DNM1*	dynamin 1	enzyme	-6.623	4.020E-03	0.133
76	*HBA1/HBA2*	hemoglobin subunit alpha 2	transporter	-6.558	9.540E-04	0.081
77	*WDR61*	WD repeat domain 61	other	-6.552	9.740E-03	0.192
78	*CYTH2*	cytohesin 2	other	-6.374	6.880E-03	0.166
79	*ISLR*	immunoglobulin superfamily containing leucine rich repeat	other	-6.159	1.070E-03	0.083
80	*BGN*	Biglycan	other	-6.158	1.160E-04	0.038
81	*TBC1D9B*	TBC1 domain family member 9B	other	-6.146	5.440E-03	0.150
82	*KLK11*	kallikrein related peptidase 11	peptidase	-5.992	2.640E-05	0.032
83	*NDUFS2*	NADH:ubiquinone oxidoreductase core subunit S2	enzyme	-5.760	9.890E-04	0.081
84	*ACOT1*	acyl-CoA thioesterase 1	enzyme	-5.732	5.860E-03	0.156
85	*RPS16*	ribosomal protein S16	other	-5.663	5.190E-04	0.065
86	*CCDC85B*	coiled-coil domain containing 85B	other	-5.655	4.340E-04	0.064
87	*MAP1LC3A*	microtubule associated protein 1 light chain 3 alpha	other	-5.542	8.650E-03	0.184
88	*ARL6IP4*	ADP ribosylation factor like GTPase 6 interacting protein 4	other	-5.529	9.630E-03	0.191
89	*ZNF358*	zinc finger protein 358	transcription regulator	-5.485	3.630E-03	0.129
90	*KRT2*	keratin 2	other	-5.484	9.530E-04	0.081
91	*GJB3*	gap junction protein beta 3	transporter	-5.454	9.220E-03	0.189
92	*RPL39*	ribosomal protein L39	other	-5.442	2.660E-04	0.050
93	*DMKN*	Dermokine	other	-5.425	8.230E-05	0.037
94	*IGFBP6*	insulin like growth factor binding protein 6	other	-5.399	1.260E-03	0.086
95	*LCE2C/LCE2D*	late cornified envelope 2D	other	-5.366	9.450E-04	0.081
96	*LY6G6C*	lymphocyte antigen 6 family member G6C	other	-5.365	4.260E-05	0.033
97	*PPDPF*	pancreatic progenitor cell differentiation and proliferation factor	other	-5.360	1.680E-05	0.032
98	*S100A16*	S100 calcium binding protein A16	other	-5.334	9.920E-03	0.193
99	*SCAMP4*	secretory carrier membrane protein 4	other	-5.266	4.040E-03	0.133
100	*NOP53*	NOP53 ribosome biogenesis factor	other	-5.102	3.280E-05	0.032

Blue molecules are molecules that have previously been mentioned in psoriasis, either their mRNA or their proteins based on QIAGEN Knowledge Base as of October 2021.
*Molecule types are classified into complex, cytokine, enzyme, fusion gene/product, G-protein coupled receptor, group, growth factor, ion channel, kinase, ligand-dependent nuclear receptor, mature microRNA, peptidase, phosphatase, transcription regulator, translation regulator, transmembrane receptor and transporter. None of these were classified as other. DEGs, differentially expressed genes; FC, fold change.

1.3. Disease associations and biological functions

Supplementary Table S3. The psoriasis-associated genes with their expression (upregulation or downregulation) in peripheral edge of lesional skin.

	Gene symbol	Gene title	Molecule type*	Log2FC	*p*-value	FDR
			**Upregulated DEGs**			
1	*A2M*	alpha-2-macroglobulin	transporter	15.982	7.38E-04	0.072
2	*HERC6*	HECT and RLD domain containing E3 ubiquitin protein ligase family member 6	enzyme	14.724	1.41E-03	0.089
3	*DEFB4A/ DEFB4B*	defensin beta 4A	other	14.690	3.70E-03	0.130
4	*HRNR*	Hornerin	other	14.597	5.63E-03	0.153
5	*IL36A*	interleukin 36 alpha	cytokine	14.572	9.84E-03	0.193
6	*SPRR2C*	small proline rich protein 2C (pseudogene)	other	12.058	8.32E-03	0.181
7	*TWF1*	twinfilin actin binding protein 1	kinase	10.854	7.50E-03	0.172
8	*TNIP3*	TNFAIP3 interacting protein 3	other	10.794	3.99E-03	0.132
9	*SERPINB4*	serpin family B member 4	other	10.691	1.46E-03	0.090
10	*CXCL8*	C-X-C motif chemokine ligand 8	cytokine	10.681	1.49E-03	0.091
11	*ADAR*	adenosine deaminase RNA specific	enzyme	9.663	1.10E-03	0.084
12	*S100A7A*	S100 calcium binding protein A7A	other	9.559	1.62E-03	0.094
13	*DBN1*	drebrin 1	other	8.059	9.61E-05	0.037
14	*SERPINB3*	serpin family B member 3	other	7.630	9.26E-03	0.189
15	*DEFB103A/ DEFB103B*	defensin beta 103B	other	7.624	2.60E-03	0.113
16	*ALDH1A3*	aldehyde dehydrogenase 1 family member A3	enzyme	6.628	3.27E-03	0.124
17	*DSC2*	desmocollin 2	other	6.595	3.70E-03	0.130
18	*SLC6A14*	solute carrier family 6 member 14	transporter	6.106	3.93E-03	0.132
19	*KRT6A*	keratin 6A	other	5.740	3.99E-03	0.132
20	*TCN1*	transcobalamin 1	transporter	5.702	5.07E-03	0.146
21	*GJB2*	gap junction protein beta 2	transporter	5.468	4.21E-03	0.135
22	*LCE3C*	late cornified envelope 3C	other	5.451	5.86E-03	0.156
23	*SOD2*	superoxide dismutase 2	enzyme	5.415	3.82E-03	0.131
24	*KRT6C*	keratin 6C	other	5.310	6.56E-03	0.163
25	*PI3*	peptidase inhibitor 3	other	5.139	2.08E-04	0.045
26	*RSAD2*	radical S-adenosyl methionine domain containing 2	enzyme	5.056	7.78E-03	0.175
27	*S100A8*	S100 calcium binding protein A8	other	4.944	7.95E-03	0.177
28	*S100A9*	S100 calcium binding protein A9	other	4.722	7.87E-03	0.176
29	*KRT16*	keratin 16	other	4.675	9.33E-03	0.189
30	*IFI44L*	interferon induced protein 44 like	other	4.571	9.56E-03	0.190
31	*SPRR2A*	small proline rich protein 2A	other	4.512	9.73E-03	0.192
32	*IFI44*	interferon induced protein 44	other	4.398	9.86E-03	0.193
33	*PLAT*	plasminogen activator, tissue type	peptidase	4.388	9.94E-03	0.193
34	*KLK7*	kallikrein related peptidase 7	peptidase	4.224	1.54E-03	0.092
35	*LCN2*	lipocalin 2	transporter	4.182	8.59E-04	0.077
36	*HAL*	histidine ammonia-lyase	enzyme	4.097	3.68E-03	0.129
37	*TXNIP*	thioredoxin interacting protein	other	3.996	2.77E-03	0.115
38	*HIF1A*	hypoxia inducible factor 1 subunit alpha	transcription regulator	3.970	6.08E-04	0.068
39	*KLK6*	kallikrein related peptidase 6	peptidase	3.927	5.41E-03	0.150
40	*NAMPT*	nicotinamide phosphoribosyltransferase	cytokine	3.919	4.05E-03	0.133
41	*IL36G*	interleukin 36 gamma	cytokine	3.897	4.74E-03	0.142
42	*TRIM22*	tripartite motif containing 22	transcription regulator	3.779	6.08E-03	0.159
43	*APOL6*	apolipoprotein L6	transporter	3.776	8.59E-03	0.184
44	*DSG3*	desmoglein 3	other	3.599	6.75E-03	0.165
45	*ALDH3A2*	aldehyde dehydrogenase 3 family member A2	enzyme	3.457	6.56E-03	0.163
46	*KRT6B*	keratin 6B	other	3.384	6.26E-03	0.161

47	*STAT1*	signal transducer and activator of transcription 1	transcription regulator	3.384	3.02E-03	0.121
48	*OASL*	2'-5'-oligoadenylate synthetase like	enzyme	3.355	5.31E-03	0.149
49	*HSPA8*	heat shock protein family A (Hsp70) member 8	enzyme	3.271	8.68E-03	0.184
50	*OAS3*	2'-5'-oligoadenylate synthetase 3	enzyme	3.202	8.66E-03	0.184
51	*SPTLC2*	serine palmitoyltransferase long chain base subunit 2	enzyme	3.022	6.69E-03	0.164
52	*S100A7*	S100 calcium binding protein A7	other	3.002	3.85E-03	0.132
53	*MAPK6*	mitogen-activated protein kinase 6	kinase	2.918	4.11E-03	0.134
54	*SLC23A2*	solute carrier family 23 member 2	transporter	2.675	1.81E-03	0.100
55	*LCE3E*	late cornified envelope 3E	other	2.652	7.77E-03	0.175
56	*CCND2*	cyclin D2	other	2.609	6.21E-03	0.161
57	*ARHGDIA*	Rho GDP dissociation inhibitor alpha	other	2.608	6.99E-03	0.167
58	*CTSL*	cathepsin L	peptidase	2.592	7.54E-03	0.173
59	*C10orf99*	chromosome 10 open reading frame 99	cytokine	2.567	8.74E-04	0.077
60	*METAP2*	methionyl aminopeptidase 2	peptidase	2.561	3.94E-03	0.132
61	*GBP1*	guanylate binding protein 1	enzyme	2.533	8.50E-03	0.183
62	*CRABP2*	cellular retinoic acid binding protein 2	transporter	2.480	8.79E-03	0.185
63	*DSG1*	desmoglein 1	other	2.456	9.52E-03	0.190
64	*ARG1*	arginase 1	enzyme	2.416	4.60E-04	0.064
65	*PLSCR1*	phospholipid scramblase 1	enzyme	2.352	8.54E-04	0.077
66	*TGM1*	transglutaminase 1	enzyme	2.320	6.30E-03	0.161
67	*TOP1*	DNA topoisomerase I	enzyme	2.223	2.60E-03	0.113
68	*CLCA2*	chloride channel accessory 2	ion channel	2.192	1.38E-03	0.089
69	*GM2A*	GM2 ganglioside activator	enzyme	2.155	3.50E-03	0.127
70	*ADAM17*	ADAM metallopeptidase domain 17	peptidase	2.146	2.26E-03	0.107
71	*FLG*	Filaggrin	other	2.135	4.21E-03	0.135
72	*LDLR*	low density lipoprotein receptor	transporter	2.126	5.81E-03	0.156
73	*CRYBG1*	crystallin beta-gamma domain containing 1	other	2.050	1.04E-03	0.082
74	*CD47*	CD47 molecule	transmembrane receptor	2.002	1.23E-04	0.038
75	*CYCS*	cytochrome c, somatic	enzyme	1.988	4.02E-03	0.133
76	*ME1*	malic enzyme 1	enzyme	1.905	9.54E-04	0.081
77	*SYNCRIP*	synaptotagmin binding cytoplasmic RNA interacting protein	other	1.867	9.74E-03	0.192
78	*DDX58*	DExD/H-box helicase 58	enzyme	1.827	6.88E-03	0.166
79	*SQLE*	squalene epoxidase	enzyme	1.790	1.07E-03	0.083
80	*IDH3A*	isocitrate dehydrogenase (NAD (+)) 3 catalytic subunit alpha	enzyme	1.736	1.16E-04	0.038
81	*PTP4A1*	protein tyrosine phosphatase 4A1	phosphatase	1.736	5.44E-03	0.150
82	*FGFBP1*	fibroblast growth factor binding protein 1	other	1.728	2.64E-05	0.032
83	*LAMA2*	laminin subunit alpha 2	other	1.626	9.89E-04	0.081
84	*IGFBP3*	insulin like growth factor binding protein 3	other	1.600	5.86E-03	0.156
85	*KPNB1*	karyopherin subunit beta 1	other	1.541	5.19E-04	0.065
86	*SPRR2D*	small proline rich protein 2D	other	1.524	4.34E-04	0.064
			**Downregulated DEGs**			
87	*GSN*	Gelsolin	other	-16.812	1.49E-03	0.091
88	*LCE1C*	late cornified envelope 1C	other	-16.153	1.62E-03	0.094
89	*ITGB4*	integrin subunit beta 4	transmembrane receptor	-15.717	3.27E-03	0.124
90	*CRAT*	carnitine O-acetyltransferase	enzyme	-15.226	4.21E-03	0.135
91	*CCL27*	C-C motif chemokine ligand 27	other	-15.021	3.82E-03	0.131
92	*PLCH2*	phospholipase C eta 2	enzyme	-13.780	6.56E-03	0.163
93	*PKM*	pyruvate kinase M1/2	kinase	-13.594	5.31E-03	0.149
94	*CFD*	complement factor D	peptidase	-9.500	8.79E-03	0.185
95	*CYP1A1*	cytochrome P450 family 1 subfamily A member 1	enzyme	-9.340	4.60E-04	0.064
96	*LORICRIN*	loricrin cornified envelope precursor protein	other	-9.244	8.54E-04	0.077
97	*KLK11*	kallikrein related peptidase 11	peptidase	-5.992	2.64E-05	0.032
98	*KRT2*	keratin 2	other	-5.484	9.53E-04	0.081

99	*LCE2B*	late cornified envelope 2B	other	-4.689	6.28E-05	0.035
100	*CLEC3B*	C-type lectin domain family 3 member B	other	-4.139	6.36E-04	0.069
101	*APOD*	apolipoprotein D	transporter	-3.864	2.21E-03	0.105
102	*KRT1*	keratin 1	other	-3.700	3.25E-04	0.056
103	*LGALS1*	galectin 1	other	-3.681	1.03E-03	0.082
104	*WNT7B*	Wnt family member 7B	other	-3.574	4.31E-03	0.136
105	*KRT10*	keratin 10	other	-3.521	1.51E-04	0.039
106	*TUBB*	tubulin beta class I	other	-3.389	1.37E-04	0.038
107	*PSORS1C2*	psoriasis susceptibility 1 candidate 2	other	-3.373	8.36E-03	0.182
108	*CSTB*	cystatin B	peptidase	-3.325	3.26E-03	0.124
109	*APOE*	apolipoprotein E	transporter	-3.300	6.71E-03	0.164
110	*GSK3A*	glycogen synthase kinase 3 alpha	kinase	-3.142	1.57E-03	0.093
111	*TIMP2*	TIMP metallopeptidase inhibitor 2	other	-3.092	3.82E-04	0.060
112	*RARG*	retinoic acid receptor gamma	ligand-dependent nuclear receptor	-2.947	2.98E-03	0.120
113	*CDHR1*	cadherin related family member 1	other	-2.931	3.27E-03	0.124
114	*CST6*	cystatin E/M	other	-2.930	9.37E-03	0.189
115	*CTNNBIP1*	catenin beta interacting protein 1	other	-2.866	6.26E-03	0.161
116	*ID4*	inhibitor of DNA binding 4, HLH protein	transcription regulator	-2.564	4.77E-03	0.143
117	*IER2*	immediate early response 2	transcription regulator	-2.344	3.55E-03	0.128
118	*TNXB*	tenascin XB	other	-2.238	2.46E-03	0.112
119	*CALML5*	calmodulin like 5	other	-2.153	6.69E-04	0.070
120	*LAMA5*	laminin subunit alpha 5	other	-2.125	7.10E-03	0.167
121	*CAV1*	caveolin 1	transmembrane receptor	-2.121	5.43E-03	0.150
122	*RER1*	retention in endoplasmic reticulum sorting receptor 1	other	-2.106	2.64E-03	0.113
123	*SULT2B1*	sulfotransferase family 2B member 1	enzyme	-2.104	3.75E-04	0.059
124	*FKBP1A*	FKBP prolyl isomerase 1A	enzyme	-2.042	4.09E-03	0.133
125	*OTUB1*	OTU deubiquitinase, ubiquitin aldehyde binding 1	enzyme	-2.021	8.23E-03	0.180
126	*TYK2*	tyrosine kinase 2	kinase	-1.915	5.78E-03	0.156
127	*CTSK*	cathepsin K	peptidase	-1.893	4.34E-03	0.136
128	*MIF*	macrophage migration inhibitory factor	cytokine	-1.842	4.86E-03	0.144
129	*CCND1*	cyclin D1	transcription regulator	-1.827	4.37E-03	0.137
130	*IL20RB*	interleukin 20 receptor subunit beta	other	-1.788	2.68E-03	0.114
131	*SLC25A5*	solute carrier family 25 member 5	transporter	-1.642	3.22E-03	0.123
132	*IGFBP7*	insulin like growth factor binding protein 7	transporter	-1.574	6.94E-03	0.166
133	*YWHAB*	tyrosine 3-monooxygenase/tryptophan 5-monooxygenase activation protein beta	other	-1.562	7.05E-03	0.167
134	*UQCRFS1*	ubiquinol-cytochrome c reductase, Rieske iron-sulfur polypeptide 1	enzyme	-1.560	6.06E-03	0.159
135	*PKP1*	plakophilin 1	other	-1.523	2.28E-03	0.107

The information was based on QIAGEN Knowledge Base as of October 2021.

*Molecule types are classified into complex, cytokine, enzyme, fusion gene/product, G-protein coupled receptor, group, growth factor, ion channel, kinase, ligand-dependent nuclear receptor, mature microRNA, peptidase, phosphatase, transcription regulator, translation regulator, transmembrane receptor and transporter. None of these were classified as other. DEGs, differentially expressed genes; FC, fold change.

Supplementary Table S5 Lists of upregulated DEGs in each pathway or function.

GO term	Description	Associated DEGs with upregulation in peripheral edge of lesional skin
R-HSA-6809371	Formation of the cornified envelope	***AFDN***, *ALDH3A2,* ***ASH1L, BMPR2, CLIC4,*** *CRABP2,* ***DSC1,*** *DSC2, DSG1, DSG3,* ***ERCC3***, *EREG,* ***FAT1, FEM1B, FERMT1,*** *FLG,* ***FOSL2, GRHL1,*** *HIF1A, HRNR,* ***IKBKB, IQGAP1, JAG1, KDM5B, KIAA1109,*** *KLK7, KRT16, KRT6A, KRT6B, KRT6C,* ***LCE3A,*** *LCE3C, LCE3E,* ***MYO9A, NOTCH2,*** *PI3,* ***PLEC, PLOD1, PTK7, RHCG, RPTN,*** *S100A7,* ***SLC4A7, SLC7A11, SPINK5,*** *SPRR2A, SPRR2D,* ***SPRR2F,*** *STAT1, TGM1,* ***TNFRSF1A,*** *TXNIP,* ***UGCG, XDH, ZBED6***
WP3888	VEGFA-VEGFR2 signaling pathway	***AFDN, ASCC3, CLIC1,*** *CXCL8,* ***CYBB,*** *CYCS,* ***DHX29, DSC1, EEA1, EIF4G2, EPRS1, FBXW11, FHL2, GIGYF2,*** *IGFBP3,* ***INPP5K, IQGAP1, ITCH, ITGB1, JAG1, LMAN1, LRRC59, MYH11, PNP, PRRC2C, PTPN11, RICTOR, SLC7A1,*** *SOD2,* ***SSR3,*** *STAT1,* ***STAT3, STIP1,*** *TXNIP*
R-HSA-199991	Membrane trafficking	***ACBD3, ANK3, AP1M1, CD59, COPA, CTSC, DENND4C, DENND5A, EREG, GALNT2,*** *GJB2,* ***GJB6,*** *HSPA8,* ***ITSN2, KIF1B, KIF5B, KIFAP3,*** *LDLR,* ***LMAN1, MAN1A2, MAN2A1, MYO5A, PIK3C2A, PPP6C, PRKAG1, RAB27B, RAB3GAP1, RAB3GAP2, RIN2, SEC23A, SH3D19, STON2, TBC1D10A, TMF1, TRAPPC9, TRIP11, TUBB6, VPS52, WASL, YWHAZ***
R-HSA-913531	Interferon signaling	*ADAR,* ***AKAP1, CLPB, DDX21***, *DDX58,* ***EIF4G2, G3BP1,*** *GBP1,* ***GBP4, GBP5, GBP6,*** *HIF1A,* ***HLA-A,*** *IFI44, IFI44L,* ***IKBKB, ITCH,*** *KPNB1,* ***LYST, MX2, NT5C3A, NUP155,*** *OAS3, OASL, PLSCR1,* ***PTPN11, RANBP2,*** *RSAD2,* ***SAMHD1, SETD2,*** *STAT1,* ***TRAF3IP1,*** *TRIM22,* ***WDFY4, XAF1***
R-HSA-6798695	Neutrophil degranulation	***AP1M1,*** *ARG1,* ***ATP11B, CAND1, CAP1,*** *CD47,* ***CD59, CDK13, CTSC, CYBB, CYFIP1, DSC1***, *DSG1,* ***FRK, GLB1,*** *GM2A,* ***HLA-A,*** *HRNR, HSPA8,* ***HUWE1, IQGAP1,*** *KPNB1, LCN2,* ***LYZ, PLD1, PNP, PSMD12,*** *S100A7, S100A8, S100A9, SERPINB3, TCN1*
R-HSA-6799990	Metal sequestration by antimicrobial proteins	***ABCA1,*** *ADAM17, ARG1,* ***CD274,*** *CXCL8,* ***EDNRB,*** *GJB2,* ***GJB6, IKBKB,*** *IL36A, IL36G, LCN2,* ***LYZ,*** *MAPK6, PI3, S100A7, S100A7A, S100A8, S100A9, SERPINB3, SOD2,* ***THBS1, TNIP3, USP25***
GO:0002831	Regulation of response to biotic stimulus	*A2M, ADAM17, ADAR,* ***AHR***, *ARG1,* ***ASH1L, CD274,*** *CD47,* ***CD59, CTSC, DDX21,*** *DDX58,* ***DLG1, EDNRB, EP300, EREG,*** *GBP1,* ***GBP5, HLA-A, IKBKB, ITCH, JAG1, KIF5B,*** *KLK7, LDLR,* ***LYST, MYO18A, NEAT1, NOTCH2,*** *OAS3, OASL,* ***PARP14,*** *PLAT,* ***PLEC,*** *PLSCR1,* ***PRKDC, PTGER3, PTPN11, RC3H1, RC3H2, RICTOR, RNF168,*** *RSAD2, S100A8, S100A9,* ***SAMHD1,*** *SERPINB4,* ***SERPINB9, SPINK5,*** *STAT1,* ***STAT3, SUPT6H, THBS1, TNFRSF1A, TRAF3IP1, VAV3, WASL, YES1***
GO:0048589	Developmental growth	***BMPR2, CCNB1,*** *CRABP2, DBN1,* ***EIF4G2, EP300, EREG, ITGB1, NOTCH2, DDR2, PLEC, PRKDC,*** *KLK6,* ***PTK7, PTPN11, STAT3, STK3, TIAM1, USP9X, RASAL1, IQGAP1,*** *SLC23A2,* ***KDM5B, RUFY3, CYFIP1, MACF1, NIPBL, CLIC4, GIGYF2, ITSN2,NIN, RC3H2, CHD7, DIP2B, ASPM, FMN1, ANK3, KIF5B,*** *LAMA2,* ***MYH11, MYO9A, NEB, TMF1, TOP2B, TPM1, TRIO, TTN, WASL, MAP4K4, MYCBP2, EPB41L3, NGEF, TANC2, PARD3, TAOK1, FBXO45, H1-5,*** *S100A8, S100A9, ADAM17,* ***SMARCA4, ITCH, KIAA1109, DCUN1D3, FAT1***, *GBP1,* ***PEAK1,*** *HIF1A,* ***CLCN3, PLEK, VAV3, TMEM123, EDNRB, IKBKB,*** *LDLR,* ***MAN2A1, TNFRSF1A, SPEN, HOOK3,*** *LCN2,* ***ATP8B1, PLD1,*** *TWF1,* ***DENND5A, TBC1D22A, TBC1D10A, CTSC, DLG1***
GO:0006974	Cellular response to DNA damage stimulus	***ASCC3, ATR, BCLAF1, BOD1L1, CHD1L, EP300, ERCC3, FBXO45, FEM1B, GIGYF2, H1-1, H1-3, H1-4, H1-5, HERC2, HUWE1, INO80D, JMY, MAP3K20, MCM4, NABP1, NIPBL, PDS5A, PRKDC, PTPN11, RNF168, RNF169, SAMHD1, SETD2, SETD7, SMC5, SMC6, SMCHD1, SMG1, SUPT6H, TAF1, TAOK1, THOC5, TLK2, TNFRSF1A, TOP2B, TRIP12, UBA6, UBR5, USP45, VAV3, VCPIP1, WAC***
GO:0034330	Cell junction organization	***ANK3, AFDN, AKAP11, CAST, CYFIP1,*** *DBN1,* ***DLG1,*** *DSG1,* ***EPB41L3, FBXO45, FMN1, FMNL2,*** *GJB2,* ***GJB6, GRHL1,*** *HSPA8,* ***IKBKB, IQGAP1, KIFC3, MACF1, MAP4K4, MYO9A, NGEF, PARD3, PCLO, PEAK1, PLEC, PLEK, PTPN11, PTPRK, SEPTIN11, SETD5, SLC7A11, SLK, STRN, TANC2, THBS1, TIAM1, VMP1WASL, YWHAZ***
GO:0048729	Tissue morphogenesis	*ADAM17, ALDH1A3,* ***ATP8B1, BMPR2, CCNA2, CHD7, CLIC4, CRYBG3, CYP7B1, DLG1, EXT2, FAT1, FEM1B, FERMT1, FMN1, GCNT4,*** *GJB2,* ***GJB6,*** *HECTD1, HIF1A,* ***ITGB1, JAG1, KDM5B,*** *KRT16, KRT6A, LCN2,* ***MAN2A1, MIB1, MYLK, MYO9A, NIPBL, NOTCH2, PRICKLE2, PTK7, PTPN11, RAB3GAP1, SETD2, SLC4A7, SLC7A11, SMARCA4, SMCHD1,*** *STAT1,* ***STAT3, STK3, TIAM1, TPM1, TRAF3IP1, TRIP11, TTN, USP45, VPS52***

The bold molecules have not been previously mentioned in psoriasis. DEGs, differentially expressed genes.

CORUM, comprehensive resource of mammalian protein complexes; DEG, differentially expressed gene; GO, gene ontology; ko, Kyoto Encyclopedia of Genes and Genomes (KEGG) pathway; M, canonical pathways; R-HSA, reactome gene sets; WP, WikiPathways.

Supplementary Table S6 Lists of downregulated DEGs in each pathway or function.

GO term	Description	Associated DEGs with downregulation in peripheral edge of lesional skin
GO:0030855	Epithelial cell differentiation	***ALDOC, ATF4, CAMSAP3, CASP14,*** *CAV1, CCND1,* ***CDKN1A, CLDN1,*** *CYP1A1,* ***EVPL, GPX1,*** *GSK3A,* ***HES1,*** *KRT10, KRT2,* ***LCE1A, CE1B,*** *LCE1C, LCE1D,* ***LCE1F, LCE2A,*** *LCE2B,* ***LCE6A,*** *LORICRIN,* ***NME2, PECAM1, POU3F1, PSAPL1, RAB1B, RAB25,*** *RARG,* ***SCRIB, SDC1, SH3BP1, SLC9A3R1****, SULT2B1,* ***TAGLN2, TPRN, TST, VIM,*** *WNT7B,* ***ZNF703***
R-HSA-9006934	Signaling by Receptor Tyrosine Kinases	***ACTB, AP2A1, AP2S1****, APOE,* ***ATP6AP1, ATP6V0B, ATP6V0C, ATP6V0E1, ATP6V1F, BRK1,*** *CAV1,* ***COL1A1, COL6A1, COL6A2, CSK, CYBA, DNM1, DNM2, FGFR3, HDAC3, HSPB1,*** *ID4, LAMA5,* ***MAP2K2, MAPK3, MXD4, NELFB, POLR2J, POLR2L, PTBP1, RAC1, RPS27A, SH3GL1, STUB1, UBA52, VEGFB,*** *YWHAB*
GO:0006914	Autophagy	***ATP6V0B, ATP6V0C, ATP6V0E1, CAPNS1, CPTP, CSNK2A2,*** *CTSK,* ***DAPL1, DCN, EIF4G1, FIS1, GABARAP,*** *GSK3A,* ***HSPB1,*** *ITGB4,* ***MAP1LC3A, MAP1LC3B, MAPK3, NUPR1, PINK1, PYCARD, RAB1B, RRAGA,*** *SLC25A5,* ***SNRNP70, SREBF1, STUB1, TBC1D17, TMBIM6, TOMM7, TSPO, UBXN6, ULK1, VAMP8, VPS28, VPS51, WDR45***
R-HSA-6809371	Formation of the cornified envelope	***CAPNS1, CASP14, EVPL,*** *KRT1, KRT10, KRT2,* ***KRT77, LCE1A, LCE1B,*** *LCE1C,* ***LCE1D, LCE1F, LCE2A,*** *LCE2B,* ***LCE6A,*** *LORICRIN, PKP1,* ***PKP3, PRSS8***
R-HSA-6798695	Neutrophil degranulation	***AGPAT2, ALDOA, ALDOC, ATP6V0C,*** *CALML5, CFD,* ***CST3,*** *CSTB,* ***CYBA, DEGS1, DPP7, FTH1, FTL,*** *GSN,* ***HEBP2, IST1,*** *KRT1, MIF,* ***NIT2, NME2, NPC2, PECAM1,*** *PKM, PKP1,* ***PSMD13, PSMD3, PYCARD, RAC1,*** *TIMP2,* ***TSPAN14,*** *TUBB,* ***TUBB4B, VAMP8, VAT1***
R-HSA-1474244	Extracellular matrix organization	***ADAM15, ATP2B4, BGN, CAPNS1, CD151, CD81, COL16A1, COL1A1, COL6A1, COL6A2, COL7A1, COMP, CRTAP,*** *CTSK,* ***CYBA, DCN, FBLN1, FBLN5, IGSF8,*** *ITGB4,* ***ITGB5,*** *LAMA5,* ***LAMTOR4, LTBP4, MAPK3, PCOLCE, PECAM1, RAC1, RRAGA, SDC1, SDC4, TGFBI, TIMP1,*** *TIMP2,* ***TMBIM6,*** *TNXB*
R-HSA-9711123	Cellular response to chemical stress	***ACTB, AP2A1, AP2S1,*** *APOE,* ***ARF1, ARL2, ATF4, BLMH, BOC,*** *CALML5, CAV1, CCND1,* ***CD74, CD81, CDC34, CDKN1A, CHTF8, COL1A1, COX5A, COX6B1, COX7C, COX8A, CSK, CSNK1G2, CSNK2A2, CTBP1,*** *CTNNBIP1,* ***CTSF,*** *CTSK,* ***CYBA, DCTN1, DDB2, DNM1, DNM2, EEF1G, EIF4G1, EMD, ESRRA, FBXO22, FBXO31, FGFR3,*** *FKBP1A,* ***FKBP8, GNAS, GNB2, GPX1,*** *GSK3A, GSN,* ***HDAC3, HES1, HSPB1,*** *IL20RB,* ***IST1, ITGB5, KAT2A, KLC3,*** *LAMA5,* ***LAMTOR4, MAP2K2, MAP3K11, MAPK3,*** *MIF,* ***MRC2, MZT2B, NDUFA4, NELFB, NPDC1, ORAI1,*** *OTUB1,* ***PDCD4, PFN1,*** *PKP1,* ***POLD2, POLR2J, POLR2L, PRDX2, PSMB4, PSMC5, PSMD13, PSMD3, PYCARD, RAB1B, RAC1, RPS27, RPS27A, RRAGA, RTN3, RTN4, SCRIB, SDC1, SEPTIN5,*** *SLC25A5,* ***SOD3, SPSB3, STAP2, STUB1, TAP2, TEAD3, TIMP1, TLE5,*** *TUBB,* ***TUBB4B, TUBGCP2, TXN2,*** *TYK2,* ***UBA52, UBE2J2, ULK1, VAMP8, VIM, VPS28,*** *WNT7B, YWHAB,* ***ZNF385A***
WP3888	VEGFA-VEGFR2 signaling pathway	***ALDOA, AP2A1, AP2S1, ATF4,*** *CAV1,* ***CCDC124,*** *CCND1,* ***CSK, EIF3F, EIF4G1, GPC1, GPX1, HSPB1,*** *IGFBP7,* ***ITGB5, MAP2K2, MAPK3, PFN1, PRDX2, RAC1, RACK1, RPL10A, RPL18A, RPL27, RPL5, RPL7, RPLP2, RPS11, SH3BGRL3,*** *TNXB*

The bold molecules have not been previously mentioned in psoriasis. DEGs, differentially expressed genes.

CORUM, comprehensive resource of mammalian protein complexes; DEG, differentially expressed gene; GO, gene ontology; ko, Kyoto Encyclopedia of Genes and Genomes (KEGG) pathway; M, canonical pathways; R-HSA, reactome gene sets; WP, WikiPathways.

Supplementary Table S7 Comparison of PE/UN DEGs with those reported in previous studies.

Year	Author And Reference	Sample for DEGs	Methodology	Key messages	Sample of upregulated DEGs	Sample of downregulated DEGs
2012, Jan	Jabbari et al.[1]	Lesional (LS) skin vs uninvolved (UN) skin	Whole-tissue RNA-seq	1) 1st Set of RNA-seq-derived DEGs (N=3) were compared with previously published microarray-derived DEGs (N=116).<br><br>2) The result uncovered DEGs which had not been revealed by microarray and supported IL-17 and TNF-α synergy in psoriasis.	*GPR110, SPRR2A, HEPHL1, CLEC3A, CD274, SPRR2E*	*DIRAS2, LRRC3B, DBX2, KRT25, PERF15, and DDX25*
2014, Jul	Li et al.[2]	LS skin vs healthy skin	Whole-tissue RNA-seq	This study revealed the DEGs involved in Myeloid cells, T-cells and Keratinocytes. RNA-seq can uncover low expressed transcripts.	*AKR1B10, CXCL13, CXCL8, DEFB4A, GDA, HEPHL1, LCE3A, PI3, S100A12, S100A7, S100A7A, S100A8, S100A9, SERPINB3, SERPINB4, SPRR2A, SPRR2F, TCN1, TMPRSS11D, TNIP3*	*AADACL3, PM20D1, DGAT2L6, AWAT2, AWAT1, PNPLA5, ROS1, GAL, CYP2W1, UGT3A2, C10orf129, PDE6A, BTC, TRIM55, WIF1, ELOVL3, HGD, HAO2, SYT9, LPPR5*
2014, Aug	Swindle et al.[3]	LS skin vs UN skin + data from previous microarray-derived transcripts	Whole-tissue RNA-seq (Meta-analysis)	1) The author compared RNA-seq-derived DEGs (N=3) with previously published microarray-derived DEGs (N=216).<br><br>2) Both RNA-seq and microarray uniquely identify genes as DEGs, however most	*ARL2, AZGP1, CTNNBIP1, KY, LCE2B, LOR, NFASC, PSMB2, SBF1, TRIM7*<br>(DEGs identified by RNA-seq, but not Microarray)	*COCH, ADAMTS2, ANKRD26, N6AMT1, LRRN3, SNURF, APLF, SLC25A53, TCHH, CEP290*<br>(DEGs identified by RNA-seq, but not Microarray)

				of DEGs showed a similar trend from both 2 methods.		
2016, May	Swindle et al.[4]	LS skin vs UN skin (RNA-seq-derived DEGs form previous studies)<br><br>PBMC of psoriasis patient vs PBMC of healthy individual	Whole-tissue RNA-seq (Meta-analysis)	1) Psoriasis-specific DEGs were expressed by keratinocytes and induced by IL-17A, while non-specific DEGs were expressed by inflammatory cells and induced by IFN-γ and TNF.<br><br>2) PBMC DEGs associated with MHC class I and NK cells were commonly downregulated in psoriasis and other autoimmune diseases	*ABCG4, ACRV1, ADAMDEC1, ADH7, AIM2, AKR1B10, AKR1B15, ARSF, ATP12A, BATF2, C10orf99, C6orf223, CAMP, CASP5, CATSPERB, CCL18, CCL20, CCL3, CCL4, CCL7, CCR7, CD177, CD24, CD274, CD38, CD80, CDH26, CHAC1, CHI3L2, CHRNA, CLDN17, CLEC3A, CLLU1, CLLU1OS, CMPK2, CNFN, CPLX3, CTLA4, CXCL1, CXCL10, CXCL13, CXCL17, CXCL2, CXCL6, CXCL8, CXCL9, CXCR6, CYP24A1, DEFB4A, DEFB4B, DKK4, DSC2, ENKUR, EPGN, EPHB2, FABP5, FAM83A, FCGR1A, FCGR3A, FOSL1, FOXE1, FPR1 , FPR2, FUT3, GBP6, GDA, GJB2, GJB6, GPR110, GPR15, GZMB, HABP2, HAS3, HEPHL1, HERC6, HPSE, HRH2, HRH3, HRNR, HTR3A, HYAL4, ICOS, IF127, IF144, IF16, IFNG, IGFL1, IL12B, IL17A, IL17C, IL17F, IL19, IL1B, IL20, IL26, IL36A, IL36G, IL36RN, INA, KCNJ15, KCNK10, KLHDC7B , KLK13, KLK6, KLK9 , KRT16, KRT3 , KRT6A, KRT6B, KRT6C, KYNU, LAIR2, LCE3A, LCE3C, LCE3D, LCE3E, LCN2, LILRA3,*	*AADACL3, ACADL, ACSBG1, ADAMTSL3, AGBL4, AGR3, ALDH1L1, ANKEN1, ANKRD2, ANKRD33B, AQP4, AQP7, AQP9, ATP13A5, AWAT1, AWAT2, BEST2, BTBD16, BTC, C10orf129, C1orf95, C1QL2, C5orf46, C5orf49, C9orf152, CA3, CA6, CA9, CACNA1H, CACNG4, CAMK2B, CAPN13, CBLN1, CCDC42B, CCER2, CCKBR, CCL27, CDH20, CDH4, CEACAMZ, CHAD, CHP2, CHRM1, CHRM4, CKM, CLDN23, CLDN3, CLDN8, CNTFR, COCH, Corf34, CPNE6, GABBR2, CPNE9, CRAT, CSMD1, CST6, CUX2, CYP1A1, CYP2W1, DBX2, DDC, DES, DGAT2L6, DIRAS2, DLK1, DNAH8, DOK7, EGF, ELOVL3, EMILIN3, ENHO, ERBB4, F3, FABP7, FACH, FADS2, FAM166B, FGF22, FGFBP2, FLG2, FRMD7, FSIP2, GAL, GJB4, GLDC, GPD1, GPR12, GSTA1, GSTA3, HAO2, HGD, HHATL, HMGCS2, HS3ST6, HS6ST2, HSD11B1, HSD3B1, IGFL2, IL34, IL37, KLRF2, KRT77, LCE5A, LINGO2, LINGO4, LRIT2, MACROD2, MAPK4, MAT1A, MATN4, METTL7B, MOGAT1,*

					LIPG, LRG1, LRRC55, LTF, LYPD1, MF5D2B, MMP1, MMP12, MUC4, MX1, MYO1H, NOS2, NWD2, OAS1, OAS2, OAS3, OASL, OSM, OTOP2, PAPL, PCSK1, PGBD5, PGLYRP2, PI3, PITX1, PLA2G4D, PLEKHG7, PRKCQ, PRSS22, PRSS27, PRSS53, REN, RGS1, RHCG, RND1, RRM2, RSAD2, S100A12, S100A7, S100A7A, S100A7L2, S100A8, S100A9, SAA2, SAMD9, SCNN1D, SERPINB13, SERPINB3, SERPINB4, SH2D5, SLC26A9, SLC6A14, SOST, SPRR1A, SPRR1B, SPRR2A, SPRR2B, SPRR2D, SPRR2E, SPRR2F, SPRR2G, SPRR3, SYT5, TCN1, TDO2, TGM1, TGM6, TMC5, TMEM171, TMPRSS11A, TMPRSS11D, TMPRSS4, TNIP3, TREX2, TYMP, UGT1A10, UGT1A7, UPP1, VNN1, VNN3, WNT5A, ZC3H12A	*MOGAT2, MSMB, MT4, MUC16, MUC7, MYEOV, MYH14, MYO3A, MYOC, NELL1, NEUROD2, NPAS1, NPTX1, NPY5R, ODF3L1, PCP4, PDZD7, PEBP4, PHYHIP, PLIN1, PLIN4, PLLP, PM20D1, PNCK, PNLDC1, PNPLA5, PPP1R1A, PRB1, PRB2, PRR4, PSAPL1, PSCA, PTPRQ, RBP4, RORC, ROS1, RPRML, SCGB2A1, SCGB3A1, SCUBE1, SEC14L6, SERPINA12, SERTM1, SFRP5, SLC14A1, SLC22A31, SLC26A3, SLC26A5, SLC46A2, SLC9A2, SLCO4C1, SNTB1, SP8, SPINK1, SPRR4, SRPK3, STXBP5L, SYT8, SYT9, TAC1, THRSP, TMEM132C, TMEM255A, TMEM63C, TMPRSS6, TNNC1, TNNI2, TNNT2, TNNT3, TPPP, TRDN, TRIM55, TSPAN8, TSPEAR, TTYH1, UGT3A2, UPB1, UPK1A, VGLL2, WDR72, WFDC3, WFIKKN2, WIF1, WNK2, WNT2, ZBTB16, ZP1*
2017, Dec	Swindle et al.[5]	Keratinocyte of LS skin, UN skin and healthy skin	Specific cell RNA-seq (Primary monolayer of culture keratinocyte)	1) The study identified downregulated DEGs involved in keratinocyte differentiation.<br>2) Most keratinocyte-derived DEGs expressed a similar trend to whole skin-derived DEGs.<br><br>3) Keratinocyte RNA-seq can identify more downregulated		- DEGs were found to be upregulated in whole tissue studies but downregulated in the KC study<br><br>*ACER1, C1orf68, CASP14, CST6, EGLN3, EPHB6, FLG, GJA5, IL36G, IRF4, IVL, KCNAB2, KIF1A, KRT1, KRT10, LANCL3, LCED3, LORICRIN, LSP1, MYH14,*

				DEGs than whole tissue RNA-seq.<br><br>4) UN keratinocytes may have intrinsic defects in differentiation capacity.		*PDZK1IP1, PLA2G2F, PMEL, PSAPL1, PTX3, RADIL, RASD1, S100A4, S100A8, S100A9, SCG2, SERPINA12, SERPINB2, SERPINB4, SLURP1, SLURP1, SNTB1, SPRR2B, SPRR2G, SPRR3, TCEA3, TGM3, TSPAN10*
2018, Jul	Ahn et al.[6]	Whole tissue of scalp psoriasis, palmoplantar psoriasis and plaque psoriasis	Whole-tissue RNA-seq	1) Common DEGs for the scalp, palmoplantar, and plaque psoriasis include genes that function in keratinocyte activation and proliferation.<br><br>2) The expression levels of IL-17A, IFN-γ, IL-22 and common DEGs varied between subtypes.	*S100A7A, SPRR2A, SPRR2B, SERPINB4, S100A9, KRT6, C10orf99, LCE3D, LCE3E, IL36G*	
2018, Jun	Nattkemper et al. [7]	LS kin vs healthy skin in both psoriasis and atopic dermatitis	Whole-tissue RNA-seq	1) Several genes which mediated itch are unique for psoriasis or atopic dermatitis<br><br>2) several molecules are common itch mediator among psoriasis and atopic dermatitis	- DEGs involved in itch, which are unique to psoriasis *CCL20, CCL4, CCL8, CXCR1, HRH3, IL19, IL20, IL26, IL36A, IL36G, KLK8, MRGPRX3, F2RL2(PAR3), PLCG1, S100A7, S100P, TRPM8, TRPV3*	
2019, Feb	Pasquali et al. [8]	Keratinocyte from UN skin, LS skin and healthy skin	Specific cell RNA-seq (CD45 magnetic cell sorting)	Deregulated genes enriched in function involving innate immunity, type I interferon response, cell cycle and keratinization.	*IFI44, IFI44L, DDX60, TNIP3, EPGN*	*NR4A3*

2021, Apr	Kim et al. [9]	LS skin and healthy skin	Single-cell RNA-seq (Natural killer cells, T-cell subsets, dendritic cell subsets, melanocytes and keratinocytes were isolated using flow cytometry)	1) Cutaneous type 17 T cells have transcriptome profiles that differ greatly in terms of IL-17A versus IL-17F expression and IFN-γ versus IL-10 expression.<br>2) IL-23A and IL-36G are also expressed by a subset of regulatory dendritic cells (DCs).<br>3) The decreased CCL27 expression in basal keratinocytes potentially contributes to impaired CCL27-CCR10 interaction.<br><br>-The authors suggested that single-cell RNA-seq provides a novel research platform for comparing gene expression profiles of heterogeneous immune cells in various inflammatory skin conditions.	- CD4+17 expressed *IL17A* and *IL17F*<br>- *IL17F* was undetected in CD8+17 cells<br>- Fifty percent of CD8+17 and 13.5% of CD4+17 expressed *IFNG*<br>- 5.8% of CD4+17 expressed *IL10*<br>- *IL10* was undetected in CD8+17 cells<br>- Mature DCs<br>*IL23, IL36G*<br>- semi mature DCs (regulatory DCS)<br>*IL10, IL23, IL36G*	- KC of stratum basale<br>***CCL27***

The red molecules were upregulated, whereas the ***green molecules*** were downregulated in the peripheral edge of lesion.
DEGs, differentially expressed genes

1.4. Canonical pathways

Supplementary Table S8. The 7 canonical pathways with their ratio.
They were calculated using a statistically significant *p*-value overlap < 0.05, Fisher's Exact Test and |z-score| ≥ 2.

Ingenuity Canonical Pathways	-log(*p*-value)	Ratio	z-score
eIF2 Signaling	3.25E+01	2.99E-01	-5.096
Coronavirus Pathogenesis Pathway	1.60E+01	2.53E-01	3.452
NER Pathway	4.18E+00	1.55E-01	-2.84
HER-2 Signaling in Breast Cancer	3.40E+00	1.13E-01	2.132
Role of IL-17A in Psoriasis	3.33E+00	3.57E-01	2.236
Oxidative Phosphorylation	2.88E+00	1.28E-01	-3.207
LXR/RXR Activation	2.45E+00	1.16E-01	-2.530

Ratio = mapped DEGs divided by total molecules in given pathway

1.5. Upstream regulator analysis

Supplementary Table S9. Upstream regulator analysis of peripheral edge of lesional skin.

Potential regulator	Molecule type	Predicted activity in PR skin	Log2 FC	Activation Z-score	*p*-value overlap	Co-regulator	Number of DEG targets
LARP1	translation regulator	Activated		7.42	3.290E-50	0	55
MYCN	transcription regulator	Inhibited		-5.17	8.960E-33	11	367
MLXIPL	transcription regulator	Inhibited		-6.05	2.080E-32	0	55
MYC	transcription regulator	Inhibited		-4.39	1.840E-28	19	462
OSM	cytokine	Activated		5.18	4.100E-19	16	461
RICTOR	other	Activated	2.238	6.23	3.270E-18	15	406
ESR1	ligand-dependent nuclear receptor	Activated		3.04	9.380E-15	18	525
IFNG	cytokine	Activated		2.21	4.240E-10	14	434
TNF	cytokine	Activated		2.49	2.090E-08	16	483
CD24	other	Activated		3.99	3.800E-08	7	135
VCAN	other	Activated		4.07	4.980E-08	13	216
SIRT1	transcription regulator	Inhibited		-2.70	1.500E-07	18	453
IFNA2	cytokine	Activated		3.38	2.570E-07	20	503
IL1B	cytokine	Activated		2.40	7.400E-07	17	436
IL22	cytokine	Activated		3.24	3.140E-06	17	403
TNIP1	other	Inhibited		-3.27	5.970E-06	0	11
RNASEH1	enzyme	Inhibited		-2.00	7.620E-06	0	4
EHF	transcription regulator	Activated		2.50	9.740E-06	0	17
ROCK2	kinase	Activated		2.16	9.980E-06	0	12
IRGM	enzyme	Inhibited		-2.88	9.980E-06	0	12
STAT1	transcription regulator	Activated	3.384	2.75	1.110E-05	17	452
Irgm1	other	Inhibited		-3.64	1.120E-05	11	203
IRF7	transcription regulator	Activated		3.34	1.220E-05	13	220
CDK4/6	group	Activated		2.52	1.660E-05	0	14
IFNAR1	transmembrane receptor	Activated		2.30	2.110E-05	17	411
DDX21	enzyme	Inhibited	2.52	-2.00	3.650E-05	0	4
IFNL1	cytokine	Activated		3.79	3.860E-05	15	448
Interferon alpha	group	Activated		3.43	4.670E-05	15	406
IL17C	cytokine	Activated		2.79	5.180E-05	9	235
SIRT7	enzyme	Inhibited		-2.40	6.940E-05	0	6
PTGER2	G-protein coupled receptor	Activated		2.71	9.240E-05	20	310
CAB39L	kinase	Inhibited		-2.99	1.220E-04	5	237

IL1A	cytokine	Activated		2.01	1.390E-04	14	377
PAF1	other	Activated		2.33	1.960E-04	0	10
Firre	other	Inhibited		-3.61	3.340E-04	0	13
PLA2G2E	enzyme	Inhibited		-2.22	3.600E-04	0	5
NGLY1	enzyme	Inhibited		-2.80	3.770E-04	0	8
NFKBIZ	transcription regulator	Activated		2.58	3.840E-04	11	277
IFN Beta	group	Activated		2.76	3.910E-04	17	406
CCND1	transcription regulator	Activated	-1.827	2.29	4.910E-04	18	421
miR-30c-5p (and other miRNAs w/seed GUAAACA)	mature microRNA	Inhibited		-2.02	5.690E-04	0	18
NKX2-3	transcription regulator	Inhibited		-2.14	6.330E-04	0	24
SEL1L	other	Inhibited		-2.71	1.030E-03	0	13
STK11	kinase	Inhibited		-2.39	1.420E-03	18	426
HELLS	enzyme	Activated		2.00	1.450E-03	0	5
NUPR1	transcription regulator	Inhibited	-2.183	-4.01	1.510E-03	0	43
IL36A	cytokine	Activated	14.572	2.60	2.010E-03	0	7
CAT	enzyme	Activated		2.20	2.130E-03	20	414
IRF5	transcription regulator	Activated		3.26	2.160E-03	13	251
RNY3	other	Activated		2.45	2.390E-03	0	6
IRF3	transcription regulator	Activated		4.01	2.910E-03	16	356
TLR3	transmembrane receptor	Activated		2.41	3.360E-03	15	376
HSP90B1	other	Inhibited		-2.33	3.500E-03	0	9
CARM1	transcription regulator	Inhibited		-2.17	3.830E-03	18	432
AKT1	kinase	Inhibited		-2.07	4.540E-03	18	475
IRF1	transcription regulator	Activated		2.69	4.930E-03	15	401
IL17R	complex	Activated		2.22	4.960E-03	0	5
ALDH1A2	enzyme	Activated		2.41	5.840E-03	12	359
ACKR2	G-protein coupled receptor	Inhibited		-2.65	6.690E-03	7	169
LMNB1	other	Inhibited		-2.00	6.900E-03	0	8
IPMK	kinase	Inhibited		-2.24	7.350E-03	0	5
GNA12	enzyme	Activated		2.20	7.600E-03	13	225
IL1RN	cytokine	Inhibited		-3.19	9.110E-03	15	287
ATF3	transcription regulator	Inhibited		-2.04	9.110E-03	19	440
TLR9	transmembrane receptor	Activated		3.06	1.040E-02	0	22
MSTN	growth factor	Inhibited		-2.23	1.080E-02	0	9
NR3C1	ligand-dependent nuclear receptor	Inhibited		-2.20	1.100E-02	0	52
IL10RA	transmembrane receptor	Inhibited		-2.33	1.420E-02	0	26
CDH11	other	Inhibited		-2.24	1.430E-02	0	5
PRKCA	kinase	Activated		2.15	1.450E-02	0	13
CPT1B	enzyme	Activated		2.30	1.820E-02	0	19
GCK	kinase	Activated		2.22	1.900E-02	0	5
Akt	group	Activated		2.30	2.100E-02	0	30
IFNB1	cytokine	Activated		2.09	2.320E-02	0	24
IL6	cytokine	Activated		2.56	2.430E-02	0	55
Ifn	group	Activated		3.26	2.710E-02	0	12
IL2	cytokine	Activated		2.18	2.900E-02	0	45
Ifn gamma	complex	Activated		2.40	3.130E-02	0	10
SP110	transcription regulator	Inhibited		-2.50	3.500E-02	0	13
ACOX1	enzyme	Activated		2.15	4.240E-02	0	13
KDM5A	transcription regulator	Activated	1.905	2.15	4.340E-02	0	12
ARID1A	transcription regulator	Inhibited		-2.37	4.630E-02	0	6
STING1	other	Activated		2.62	4.680E-02	0	7

The table provides the potential regulators and their predicted activity in peripheral edge of lesional skin using a statistically significant overlap between the dataset genes and the genes that are regulated by a potential regulator (*p*-value overlap < 0.05, Fisher's Exact Test) and absolute activation z-score (|z-score| ≥ 2)

*Molecule types are classified into complex, cytokine, enzyme, fusion gene/product, G-protein coupled receptor, group, growth factor, ion channel, kinase, ligand-dependent nuclear receptor, mature microRNA, peptidase, phosphatase, transcription regulator, translation regulator, transmembrane receptor and transporter. None of these were classified as other.

* Red molecules are identified as upregulated DEGs, whereas green molecules are identified as downregulated DEGs in perilesional skin

DEGs, differentially expressed genes; FC, fold change.

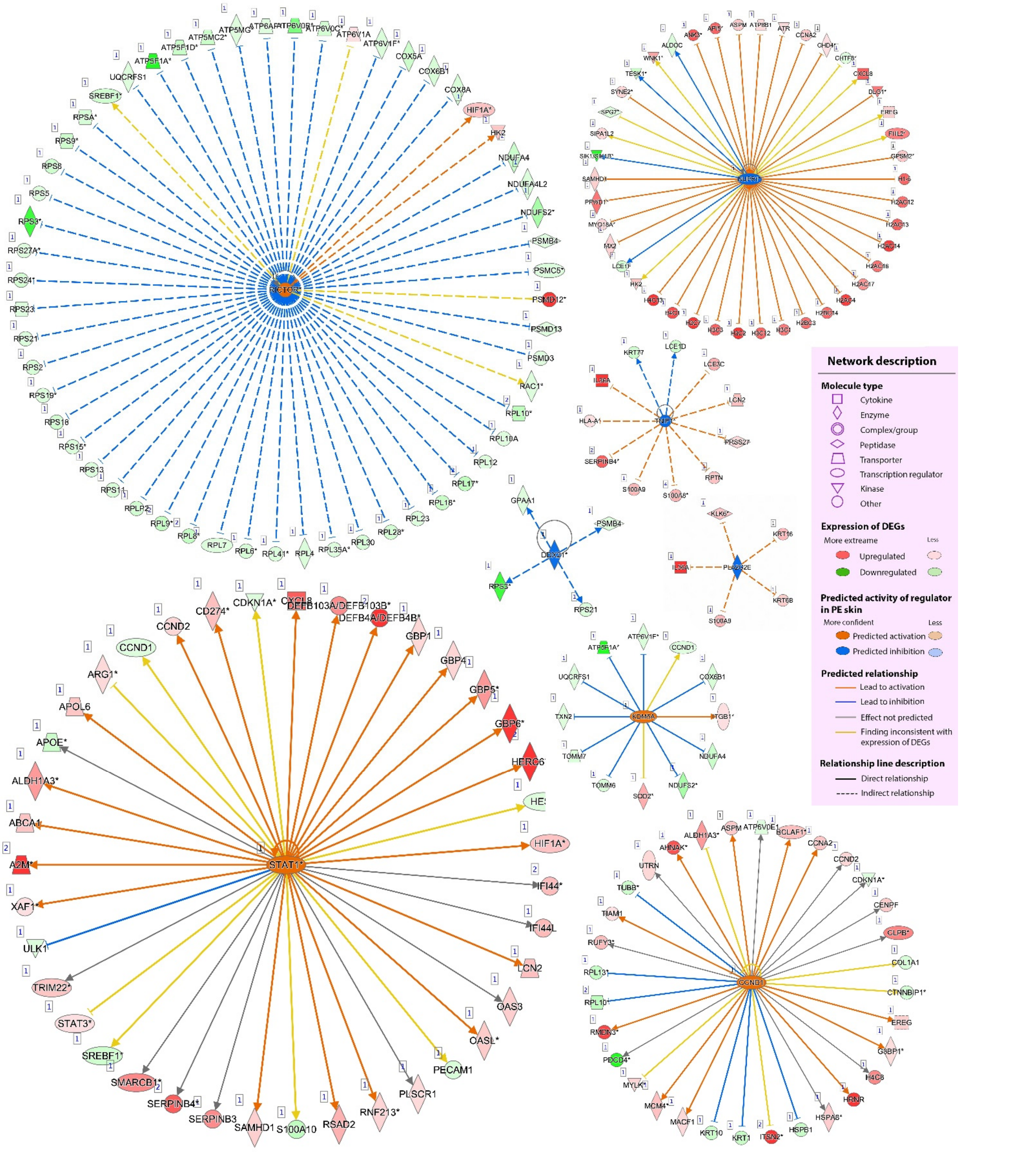

Supplementary Figure S2. Potential regulators and their relationship to target dataset's DEGs.

In each radiant, the molecule at the center is the predicted potential regulator and the outer circular molecules are the dataset's DEGs. DEGs, differentially expressed genes; PE skin, peripheral edge of lesional skin.

Supplementary Table S10. Demographic data of the patients for transcriptomic profile.

**Number**	**Age (year)**	**Sex**	**Underlying disease**	**BMI (kg/m^2)**	**PASI**	**Lesion**				**Personal history**		
						Skin	Plaque characteristic	Scalp lesion	Nail involvement	Psoriasis in family member	Alcohol drinking	Smokin g
1	23	Male	✗	20.3	12.2	✓	Instable	✓	✓	✓	✗	✗
2	39	Male	✗	20.5	35.5	✓	Instable	✗	✓	✗	✗	✗
3	57	Male	✗	24.4	12.5	✓	Instable	✓	✓	✗	✗	✗

BMI; Body mass index
PASI; Psoriasis area severity index

3. Methodology

Supplementary Table S11. Assay ID of the primers for multiplex qPCR

	Gene Symbol	Assay ID
DEGs	*IL36A*	Hs01015266_g1
	PLSCR1	Hs01062171_m1
	CXCL8	Hs00174103_m1
	SERPINB4	Hs01691258_g1
	DEFB4A/DEFB4B	Hs00175474_m1
	DEFB103A/DEFB103B	Hs04195435_g1
	S100A7	Hs00161488_m1
	S100A7A	Hs00752780_s1
	S100A8	Hs00374264_g1
	S100A9	Hs00610058_m1
Regulators	*IL17C*	Hs00171163_m1
	NFKBIZ	Hs00230071_m1
	ROCK2	Hs00178154_m1
Internal control	*GAPDH*	Hs02786624_g1

DEGs, differentially expressed gene

References

1. Jabbari, A.; Suárez-Fariñas, M.; Dewell, S.; Krueger, J.G. Transcriptional profiling of psoriasis using RNA-seq reveals previously unidentified differentially expressed genes. *J Invest Dermatol* **2012**, *132*, 246-249, doi:10.1038/jid.2011.267.
2. Li, B.; Tsoi, L.C.; Swindell, W.R.; Gudjonsson, J.E.; Tejasvi, T.; Johnston, A.; Ding, J.; Stuart, P.E.; Xing, X.; Kochkodan, J.J.; et al. Transcriptome analysis of psoriasis in a large case-control sample: RNA-seq provides insights into disease mechanisms. *J Invest Dermatol* **2014**, *134*, 1828-1838, doi:10.1038/jid.2014.28.
3. Swindell, W.R.; Xing, X.; Voorhees, J.J.; Elder, J.T.; Johnston, A.; Gudjonsson, J.E. Integrative RNA-seq and microarray data analysis reveals GC content and gene length biases in the psoriasis transcriptome. *Physiol Genomics* **2014**, *46*, 533-546, doi:10.1152/physiolgenomics.00022.2014.
4. Swindell, W.R.; Sarkar, M.K.; Liang, Y.; Xing, X.; Gudjonsson, J.E. Cross-Disease Transcriptomics: Unique IL-17A Signaling in Psoriasis Lesions and an Autoimmune PBMC Signature. *J Invest Dermatol* **2016**, *136*, 1820-1830, doi:10.1016/j.jid.2016.04.035.
5. Swindell, W.R.; Sarkar, M.K.; Liang, Y.; Xing, X.; Baliwag, J.; Elder, J.T.; Johnston, A.; Ward, N.L.; Gudjonsson, J.E. RNA-seq identifies a diminished differentiation gene signature in primary monolayer keratinocytes grown from lesional and uninvolved psoriatic skin. *Sci Rep* **2017**, *7*, 18045, doi:10.1038/s41598-017-18404-9.
6. Ahn, R.; Yan, D.; Chang, H.W.; Lee, K.; Bhattarai, S.; Huang, Z.M.; Nakamura, M.; Singh, R.; Afifi, L.; Taravati, K.; et al. RNA-seq and flow-cytometry of conventional, scalp, and palmoplantar psoriasis reveal shared and distinct molecular pathways. *Sci Rep* **2018**, *8*, 11368, doi:10.1038/s41598-018-29472-w.
7. Nattkemper, L.A.; Tey, H.L.; Valdes-Rodriguez, R.; Lee, H.; Mollanazar, N.K.; Albornoz, C.; Sanders, K.M.; Yosipovitch, G. The Genetics of Chronic Itch: Gene Expression in the Skin of Patients with Atopic Dermatitis and Psoriasis with Severe Itch. *J Invest Dermatol* **2018**, *138*, 1311-1317, doi:10.1016/j.jid.2017.12.029.
8. Pasquali, L.; Srivastava, A.; Meisgen, F.; Das Mahapatra, K.; Xia, P.; Xu Landén, N.; Pivarcsi, A.; Sonkoly, E. The Keratinocyte Transcriptome in Psoriasis: Pathways Related to Immune Responses, Cell Cycle and Keratinization. *Acta Derm Venereol* **2019**, *99*, 196-205, doi:10.2340/00015555-3066.
9. Kim, J.; Lee, J.; Kim, H.J.; Kameyama, N.; Nazarian, R.; Der, E.; Cohen, S.; Guttman-Yassky, E.; Putterman, C.; Krueger, J.G. Single-cell transcriptomics applied to emigrating cells from psoriasis elucidate pathogenic versus regulatory immune cell subsets. *J Allergy Clin Immunol* **2021**, *148*, 1281-1292, doi:10.1016/j.jaci.2021.04.021.
